# Fenoldopam to prevent acute kidney injury after major surgery—a systematic review and meta-analysis

**DOI:** 10.1186/s13054-015-1166-4

**Published:** 2015-12-25

**Authors:** Michael A. Gillies, Vivek Kakar, Robert J. Parker, Patrick M. Honoré, Marlies Ostermann

**Affiliations:** Department of Anaesthesia, Critical Care & Pain Medicine, Royal Infirmary of Edinburgh, Edinburgh, UK; Department of Critical Care, King’s College Hospital NHS Foundation Hospital, London, UK; Aintree University Hospital NHS Foundation Hospital, Department of Critical Care Medicine, Liverpool, UK; Department of ICU, Universitair Ziekenhuis Brussel, Vrije Universiteit Brussel (VUB University), Brussels, Belgium; Department of Nephrology & Critical Care Medicine, King’s College London, Guy’s & St Thomas’ Foundation Hospital, London, UK

**Keywords:** Fenoldopam, AKI, Systematic review, Meta-analysis, Acute kidney injury

## Abstract

**Background:**

Acute kidney injury (AKI) after surgery is associated with increased mortality and healthcare costs. Fenoldopam is a selective dopamine-1 receptor agonist with renoprotective properties. We conducted a systematic review and meta-analysis of randomised controlled trials comparing fenoldopam with placebo to prevent AKI after major surgery.

**Methods:**

We searched EMBASE, PubMed, meta-Register of randomised controlled trials and Cochrane *CENTRAL* databases for trials comparing fenoldopam with placebo in patients undergoing major surgery. The primary outcome was incidence of new AKI. Secondary outcomes were requirement for renal replacement therapy and hospital mortality.

**Results:**

Eighty-three publications were screened; 23 studies underwent full data extraction and scoring. Six trials were suitable for inclusion in the data synthesis (total of 507 subjects undergoing cardiovascular surgery, partial nephrectomy, liver transplant surgery). Five studies were rated at high risk of bias. Data on post-operative incidence of AKI were available in five of the six trials (total of 471 patients) but definitions of AKI varied between studies. Of the 238 patients receiving fenoldopam, 45 (18.9 %) developed AKI compared to 62 (26.6 %) of the 233 patients who received placebo (*p* = 0.004, *I*^2^ = 0 %; random-effects model odds ratio 0.46, 95 % confidence interval 0.27–0.79). In patients treated with fenoldopam, there was no difference in renal replacement therapy (n = 478; *p* = 0.11, *I*^2^ = 47 %; fixed-effect model odds ratio 0.27, 95 % confidence interval 0.06–1.19) or hospital mortality (*p* = 0.60, *I*^2^ = 0 %; fixed-effect model odds ratio 1.0, 95 % confidence interval 0.14–7.37).

**Conclusions:**

In this analysis, peri-operative treatment with fenoldopam was associated with a significant reduction in post-operative AKI but it had no impact on renal replacement therapy or hospital mortality. Equipoise remains for further large trials in this area since the studies were conducted in three types of surgery, the majority of studies were rated at high risk of bias and the criteria for AKI varied between trials.

**Electronic supplementary material:**

The online version of this article (doi:10.1186/s13054-015-1166-4) contains supplementary material, which is available to authorized users.

## Background

Acute kidney injury (AKI) is an important complication of high-risk surgery and is associated with increased morbidity, mortality and healthcare costs [1–3]. Current management strategies are largely preventative and consist of avoidance of nephrotoxins, optimisation of haemodynamics (including arterial pressure) and correction of volume depletion. At present there are no therapeutic agents that have demonstrated efficacy in preventing or treating post-operative AKI.

Fenoldopam is a short-acting dopamine-1 receptor agonist, licensed for the treatment of hypertension. It decreases systemic vascular resistance while simultaneously increasing renal blood flow in patients with normal renal function and those with chronic kidney disease (CKD) [4]. It has been investigated in off-label studies as a potential agent to protect renal function during the peri-operative period [5, 6]. In contrast to dopamine, it has no α- or β-receptor activity. Data from experimental AKI models suggest that it also has anti-inflammatory effects independent of its vasodilatory action [7].

At least three meta-analyses have suggested that fenoldopam may reduce the incidence of AKI, reduce the requirement for renal replacement therapy (RRT), and reduce mortality during critical illness and in patients undergoing cardiovascular interventions [8–10]. However, some of the studies included in these reviews were either non-randomised, studies with an active comparator, or trials performed in non-surgical patients.

Guidance on the role of fenoldopam in surgical and critically ill patients is conflicting. In 2010, the European Society of Intensive Care Medicine Working Group for Nephrology published recommendations for the prevention of AKI and suggested that the prophylactic use of fenoldopam should be considered in patients at high risk of AKI undergoing cardiovascular surgery [11]. Two years later, the Kidney Disease Improving Global Outcomes (KDIGO) expert committee released a new guideline and advised against the use of fenoldopam to prevent or treat AKI in any context [12]. New data produced in 2010–2012 may have contributed to the change in guidance.

Our aim was to review the existing data for fenoldopam as a renoprotective agent in the peri-operative setting and to perform a meta-analysis and systematic review of all existing randomised controlled trials (RCTs) comparing fenoldopam versus placebo in adult patients undergoing any type of major surgery.

## Methods

### Search strategy

We searched EMBASE, PubMed, meta-Register of RCTs (mRCT) and Cochrane CENTRAL databases using the following pre-agreed search strategy: (fenoldopam OR Corlopam) [tiab] AND rand*[tiab]. The search was limited to RCTs in adult subjects. The bibliographies of evaluable studies and other selected papers were hand searched. Authors of published studies were also contacted for more data as needed. Experts were contacted and asked if they were aware of other studies not identified by our search strategy. Two authors (VK and RJP) performed the literature search independently. Any disparities were resolved by the consensus of all authors. The search strategy and analysis were performed as per the Preferred Reporting Items for Systematic Review and Meta-Analysis (PRISMA) statement 2009 [13].

### Study selection

Two investigators (VK and RJP) independently examined the abstracts of studies identified by the literature search. The following criteria were used for inclusion in the analysis: RCTs (blinded or unblinded) published between 1970 and 2015; trials comparing fenoldopam versus placebo for prevention of AKI; inclusion of patients undergoing major surgery (elective or emergency); and complete data available on any of the outcomes (Table 1). The exclusion criteria were: duplicate publications; studies not published in English; crossover studies; non-human experimental studies; or lack of complete or sufficient data to perform a meta-analysis of targeted outcomes. Two investigators (MAG and MO) then independently reviewed the full studies and assessed compliance with the pre-agreed selection criteria. Any disagreements were reviewed and resolved by a third author (PMH).

**Table 1:** Specification of the research question applying a PICO (population, intervention, comparison and outcome) model

Population	Intervention	Comparison	Outcomes
Adult patients (≥18 years) undergoing any type of major surgery	Treatment with fenoldopam in the peri-operative period	Placebo	Incidence of AKI Requirement for RRT post-operatively Hospital mortality Side effects (hypotension, need for catecholamine treatment)

*AKI* acute kidney injury, *RRT* renal replacement therapy

The Cochrane Collaboration Risk of Bias tool was used to assess the internal validity of included trials [14]. This tool consists of six domains (allocation concealment, random sequence generation, blinding, selective outcome reporting, incomplete outcome data, and other sources of bias). Each domain is rated low risk, unclear risk, or high risk. If one or more individual domains were considered as having a high risk of bias, the overall score was assessed as having a high risk of bias. The overall risk of bias was rated low only if all components were assessed as having a low risk of bias.

### Data extraction

Two investigators (PMH and MO) independently extracted the following information from each eligible article: study design (including patient selection and treatment allocation), population, clinical setting, dosage and duration of fenoldopam treatment, incidence of new AKI, requirement for RRT, hospital mortality and reported complications. If the necessary data could not be extracted from the publication, the original authors were contacted.

### Outcomes

The primary outcome was incidence of new AKI (as defined by the author). Secondary outcomes were requirement for RRT and hospital mortality. If data on mortality was not reported, data on AKI or RRT were used; conversely, if only data on mortality were available then this was used. A priori, a decision was made to carry out a subgroup analysis on patients undergoing cardiac surgery.

### Statistical analysis

Statistical analysis was performed using Review Manager (RevMan) v5.3, software used for preparing and maintaining Cochrane Reviews. Between-study statistical heterogeneity was assessed by χ^2^ test and *I*^2^ test; values of the index of 25, 50, and 75 % indicated the presence of low, moderate, and high between-trial heterogeneity, respectively. A *p*-value of 0.1 was considered to indicate statistical significance of heterogeneity. The funnel plot method was used to estimate potential publication bias for any of the outcomes, either primary or secondary. Dichotomous outcomes were expressed as a difference of proportions (odds ratio (OR) with 95 % confidence interval (CI)). If no significant heterogeneity was noted, the fixed-effect model (FEM) analysis using the Mantel-Haenszel method was performed. Otherwise, the Simonian-Laird method was used to present the results of the random-effects model (REM) analysis.

## Results

### Study selection

**Fig. 1:**
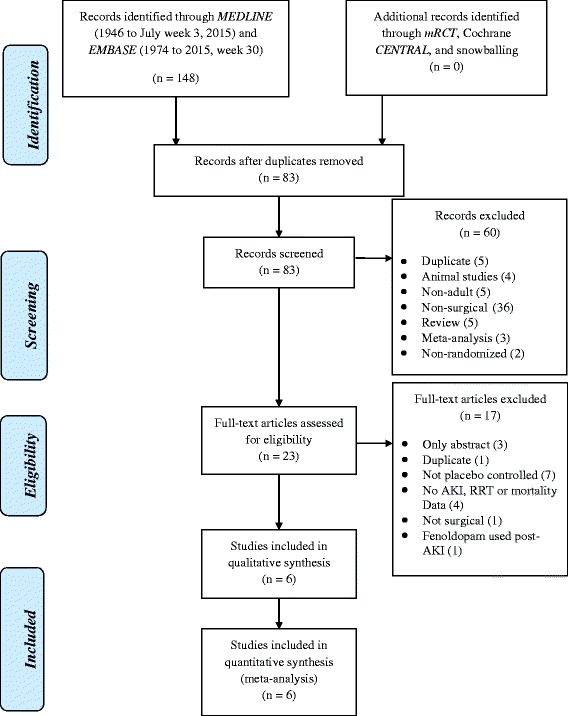
PRISMA flow diagram detailing search strategy and identification of studies used in data synthesis. *AKI* acute kidney injury, *RRT* renal replacement therapy, *mRCT* metaRegister of Controlled Trials

Figure 1 outlines the process for literature searching and study selection. Eighty-three non-duplicate publications were screened; 23 studies underwent full data extraction and scoring. Six trials were suitable for inclusion in the data synthesis (total of 507 subjects) (Table 2). Five trials were rated at high risk of bias (Table 3).

**Table 2:** Summary of included studies

Author	Time of study	Type of surgery	Number of patients (F/P)	Age (F/P)	Dose and duration of fenoldopam	Inclusion of CKD patients	Definition of AKI	Reported outcomes	Reported side effects
O’Hara et al. [19]	Nov 2002 to April 2010	Partial nephrectomy in patients with single kidney	77 (43/34)	59/59 (mean)	0.1 μg/kg/min for 24 h	Not reported	RIFLE criteria or change in GFR	AKI RRT data provided separately by authors	None reported
Ranucci et al. [17]	Sept 2008 to March 2009	Elective cardiac surgery with expected CPB time ≥90 mins	80 (40/40)	65/64 (mean)	0.1 μg/kg/min from pre-operative to 12 h post-opertive	Yes (including 2 patients on chronic dialysis)	Peak Cr >2 mg/dL and 100 % rise from baseline for >24 h	AKI 30-day mortality	Stroke, mechanical ventilation, sternal wound infection, re-exploration
Barr et al. [15]	May 2002 to Jan 2006	Elective or emergency cardiac surgery in patients with pre-operative CrCl ≤40 ml/min	38 (19/19)	77.2/72.4 (mean)	0.1 μg/kg/min for 48 h	Yes (CKD was an inclusion criterion)	Difference in CrCl as per Cockcroft-Gault formula between pre-operative and post-operative day 3	CrCl RRT Hospital mortality	Use of vasopressors and inotropes
Cogliati et al. [16]	Not reported	High-risk cardiac surgery	193 (95/98)	70.3/69.6 (mean)	0.1 μg/kg/min started before surgical incision until 24 h post-operative	Yes	Serum Cr rise to >2 mg/dl with a rise of ≥0.7 mg/dl from pre-operative to post-operative	AKI RRT	None reported
Biancofiore et al. [18]	Jan 2001 to July 2003	Liver transplant surgery	92 (46/46)	45/51 (median)	0.1 μg/kg/min from induction until 96 h post-operative	Yes (provided pre-operative Cr ≤1.5 mg/dL)	Serum Cr rise to >1.5 mg/dl or doubling of Cr or need for RRT	AKI RRT Hospital mortality	None reported
Halpenny et al. [5]	Not reported	Elective aortic surgery with infra-renal cross-clamping	27 (14/13)	70/69 (mean)	0.1 μg/kg/min from surgical incision until release of aortic cross-clamp	No	Not specifically defined	AKI	Blood loss, MAP and heart rate

*AKI* acute kidney injury, *CKD* chronic kidney disease, *CPB* cardiopulmonary bypass, *Cr* creatinine, *CrCl* creatinine clearance, *F* Fenoldopam, *GFR* glomerular filtration rate, *MAP* mean arterial blood pressure, *P* placebo, *RIFLE* Risk–Injury–Failure–Loss–End-stage renal disease, *RRT* renal replacement therapy

**Table 3:** Quality summary: authors’ assessment of risk of bias in randomised controlled trials included in meta-analysis

Author	Random sequence generation	Allocation concealment	Blinding of participants, personnel and outcome assessors	Incomplete outcome data	Selective outcome reporting	Commercial involvement	Other sources of bias	Overall assessment
O’Hara et al. [19]	Low risk	Low risk	Low risk	Low risk	Low risk	Low risk	Low risk	Low risk
Ranucci et al. [17]	Low risk	Low risk	Low risk	Low risk	Low risk	Low risk	High risk^a^	High risk
Barr et al. [15]	Unclear	Low risk	Low risk	Low risk	Low risk	Low risk	Low risk^a^	High risk
Cogliati et al. [16]	Low risk	Low risk	Low risk	Low risk	Low risk	Unclear	High risk^a^	High risk
Biancofiore et al. [18]	Low risk	Low risk	Low risk	Low risk	Low risk	Low risk	High risk^a^	High risk
Halpenny et al. [5]	Unclear	Unclear	Low risk	Low risk	Low risk	Unclear	High risk^b^	High risk

Risk of bias was judged by the authors to be high, unclear or low according to the Cochrane Collaboration’s risk of bias assessment tool

^a^High risk due to potential imbalance in number of patients with pre-existing chronic kidney disease

^b^High risk because the definition of AKI was not provided

### Characteristics of included studies

Three of the six included trials were performed in patients undergoing cardiac surgery [15–17]. The remaining three studies were undertaken in patients having aortic surgery [5], transplant surgery [18] or a partial nephrectomy [19] (Table 2). Funnel plot of studies used in the primary outcome analysis indicated no evidence of publication bias (Additional file 1).

### Primary outcome

**Fig. 2:**
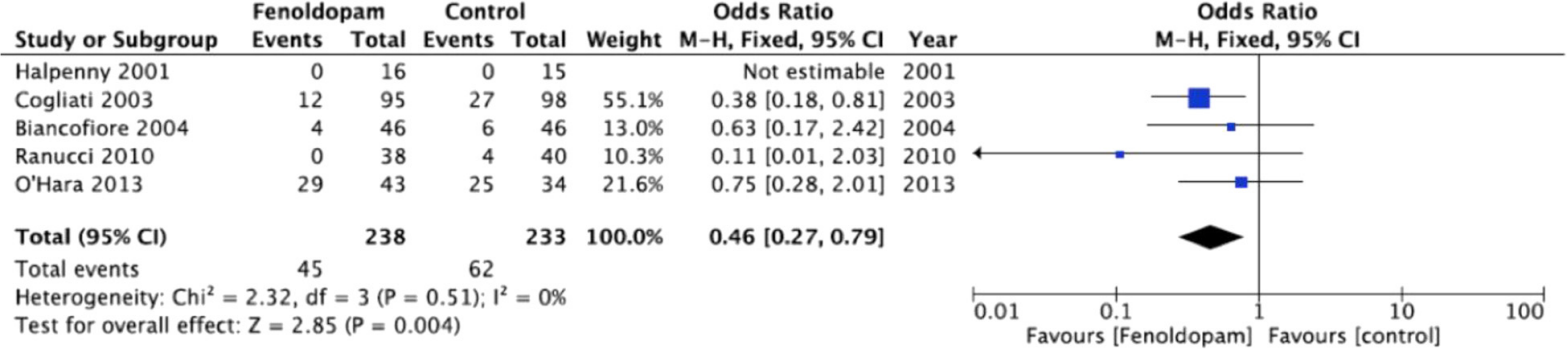
Forest plot of incidence of new acute kidney injury (as defined by authors). *CI* confidence interval, *M-H* Mantel-Haenszel

Data on post-operative incidence of new AKI were available in five of the six trials included in the analysis (total of 471 patients). In one study no patient developed AKI [5]. Significant reduction in AKI was observed in patients receiving fenoldopam (Fig. 2). Of 238 patients receiving fenoldopam, 45 (18.9 %) developed AKI compared to 62 (26.6 %) of 233 patients who received placebo (*p* = 0.004, *I*^2^ = 0 %; REM OR 0.46, 95 % CI 0.27–0.79). Three of these studies (n = 304) were performed in cardiac surgery patients [15–17]. When restricted to this cohort, significant reduction in author-defined AKI was also found between the group receiving fenoldopam and the control group (*p* = 0.003, *I*^2^ = 0 %; FEM OR 0.34, 95 % CI 0.16–0.69) (Additional file 2).

### Secondary outcomes

Data on new requirement for RRT in the post-operative period were available in five of the six RCTs (total of 478 patients) [15–19]. Of 241 patients in the fenoldopam group, 2 (0.8 %) were treated with RRT post-operatively compared to 12 (4.5 %) of the 237 patients treated with placebo. The reduction in incidence of the need for RRT reported in the treatment arm was not significant (*p* = 0.11, *I*^2^ = 47 %; FEM OR 0.27, 95 % CI 0.06–1.19) (Additional file 3).

**Fig. 3:**
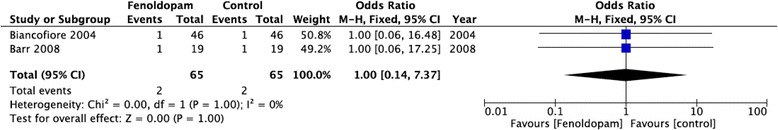
Forest plot for hospital mortality. *CI* confidence interval, *M-H* Mantel-Haenszel

Hospital mortality data were included in only two of the six included RCTs, a total of 130 patients [15, 19]. Of the 65 patients receiving fenoldopam, 2 (3.1 %) died compared to 2 (3.1 %) of 65 patients receiving placebo. There was no difference in hospital mortality between the fenoldopam and placebo group (*p* = 0.60, *I*^2^ = 0 %; FEM OR 1.0, 95 % CI 0.14–7.37) (Fig. 3).

## Discussion

The main finding of this analysis is that peri-operative administration of fenoldopam to patients undergoing cardiovascular surgery, partial nephrectomy or liver transplant surgery significantly reduced the development of AKI but did not significantly alter the requirement for RRT or hospital mortality. Whether the conclusions can be translated to other types of major surgery is unclear.

AKI is common in hospitalised patients, and influences both short- and long-term mortality. The recent multi-national Acute Kidney Injury – Epidemiologic Prospective Investigation (AKI-EPI) study demonstrated that AKI occurred in more than half of intensive care unit (ICU) patients (57 %) [20]. Amongst patients admitted after surgery, 53 % developed post-operative AKI. A study in patients undergoing cardiac surgery showed that those who developed AKI post-operatively had a significantly increased risk of dying for up to 10 years after surgery, even if renal function had recovered at the time of hospital discharge [21]. Similar data have also been reported for patients undergoing other major surgical procedures [22]. Hence, strategies to reduce or mitigate the incidence of peri-operative AKI are of high interest to clinicians.

The exact pathophysiology of AKI is not fully understood but includes haemodynamic factors, dysregulation of the renal microcirculation, inflammatory processes, mitochondrial dysfunction and bio-energetic disturbance, and exposure to toxic substances. Peri-operative hypoperfusion is an important risk factor for AKI following surgery, especially in combination with hypovolaemia [23]. It has been hypothesised that fenoldopam may be able to potentially reverse renal hypoperfusion, and hence prevent or resolve AKI. In addition, fenoldopam may also have anti-inflammatory effects independent of its vasodilatory action [7].

Landoni et al. conducted three separate meta-analyses exploring the role of fenoldopam [8–10]. The first meta-analysis focussed on the effect of fenoldopam in critically patients who had or were at risk of AKI [8]. Sixteen RCTs were included (total of 1290 patients). The analysis concluded that fenoldopam significantly reduced the development of AKI, need for RRT, length of stay (LOS) in ICU and hospital, and mortality. However, the analysis was limited by the inclusion of both surgical and non-surgical critically ill patients (i.e. sepsis), only 10 of the included studies were placebo-controlled, most included studies were of low quality, and the indications for starting RRT were non-uniform between studies. Subsequently, they performed another meta-analysis with focus on studies in patients who underwent cardiovascular surgery [9]. They concluded that fenoldopam significantly reduced the need for RRT and was associated with a lower in-hospital mortality. Of note, of the 13 studies included in this meta-analysis, only nine were randomised, and of these only four were placebo-controlled. However, a post-hoc subgroup analysis of just the RCTs confirmed the beneficial effects of fenoldopam. Finally, the same authors completed a third meta-analysis where only placebo-controlled RCTs of fenoldopam in cardiac surgery patients were included [10]. Whilst there was a significant reduction in the development of AKI, fenoldopam did not have a statistically significant effect on RRT, or hospital and ICU LOS.

Landoni and colleagues also conducted a multi-centre, double-blind, placebo-controlled RCT to determine whether fenoldopam reduced the need for RRT in critically ill cardiac surgery patients with AKI [24]. There was no difference in RRT, ICU LOS, or mortality but a higher incidence of hypotension in patients receiving fenoldopam. While this is the largest study on the subject till date (667 patients overall), it had several limitations. The study was stopped early due to futility resulting in recruitment of only 667 patients instead of the planned 1000 patients. AKI was defined as per Risk–Injury–Failure–Loss–End-stage renal disease (RIFLE) classification but the criteria for initiating RRT were not pre-defined and left to the judgement of the treating clinicians.

To the best of our knowledge, our study is the only meta-analysis of fenoldopam use in the wider surgical population. Strengths of our study were that robust methodology was employed with an a priori analysis plan and only studies involving subjects undergoing surgery were included. All studies included were RCTs published in peer-reviewed journals. There was little statistical evidence of significant heterogeneity between studies; heterogeneity was absent in the primary outcome and in the secondary outcome of hospital mortality. Moderate heterogeneity was detected in the secondary outcome of new requirement for RRT.

However, in common with many meta-analyses, our study did have several potential sources of bias. The definition of AKI was not uniform between studies. In fact, all studies used different criteria for AKI as demonstrated in Table 2. Some investigators used very conservative criteria (e.g. peak creatinine >2 mg/dL (196 μmol/L)). None of the studies had pre-defined criteria for RRT. It is important to emphasize that our results have to be weighted on this limitation. It is certainly possible that different rates of AKI may have been observed if the authors had used current consensus criteria which define AKI by a rise in serum creatinine >26.4 μmol/L in 48 hours or less, or a 50 % rise in 7 days or less (according to the latest KDIGO consensus criteria) [12]. Furthermore, our results are based on studies that were performed in three specific types of surgery only, i.e. cardiovascular surgery, partial nephrectomy and liver transplant surgery. Whether the conclusions can be translated to other types of major surgery is unclear. It is also important to acknowledge that all included studies were underpowered. Only 473 patients were included in the meta-analysis for the primary outcome, and mortality data were only available for two studies (130 patients). Five of six studies included were deemed at high risk of bias using the Cochrane Assessment Tool [14]. The main reasons for this were a potential imbalance in the number of patients with pre-existing CKD. Finally, if fenoldopam does indeed have a beneficial effect on renal function during the peri-operative period, the optimal duration of therapy is unknown. In all studies, fenoldopam was administered at 0.1 μg/kg/min, but the duration of therapy varied from the end of surgery to 96 hours post-operatively.

In summary, there may be a role for fenoldopam in preventing AKI following high-risk surgery; however, identification of the patients most likely to benefit and the optimal dose and duration of therapy remains yet to be defined. Future well-powered studies should compare fenoldopam with placebo in a much wider patient group with normal renal function undergoing high-risk surgery. Moreover the definitions of AKI, and the indications for RRT, should also be pre-defined and be consistent with current consensus criteria.

## Conclusions

This meta-analysis suggests that peri-operative fenoldopam administration in surgical patients was associated with a reduction in AKI but there was no effect on RRT or mortality. Non-standard classification of AKI in the majority of studies included in this analysis means that these results should be interpreted with great caution. Equipoise remains for further adequately powered trials of fenoldopam in the high-risk surgical population; however, optimum dose and duration of therapy have yet to be defined.

## Key messages

- Meta-analysis of six randomised controlled trials showed that peri-operative treatment with fenoldopam was associated with a significant reduction in acute kidney injury after major surgery.
- Peri-operative treatment with fenoldopam had no impact on requirement for renal replacement therapy or hospital mortality.
- Further research is required to determine the role of fenoldopam since the studies only included cardiovascular surgery, partial nephrectomy and liver transplant surgery, the majority of studies were at high risk of bias and the criteria for acute kidney injury varied between studies.

## Additional files

Additional file 1:
**Funnel plot for primary outcome (author defined acute kidney injury).** (DOC 55 kb) Additional file 2:
**Forrest plot of acute kidney injury for cardiac surgery subgroup.** (DOC 58 kb) Additional file 3:
**Forest plot of new requirement for renal replacement therapy.** (DOC 68 kb)

## Abbreviations

AKI: Acute kidney injury
CI: Confidence interval
CKD: Chronic kidney disease
FEM: Fixed-effect model
ICU: Intensive care unit
KDIGO: Kidney Disease Improving Global Outcomes
LOS: Length of stay
OR: Odds ratio
PRISMA: Preferred Reporting Items for Systematic Review and Meta-Analysis
RCT: Randomised controlled trial
REM: Random-effects model
RIFLE: Risk–Injury–Failure–Loss–End-stage renal disease
RRT: Renal replacement therapy
