# Supplementary figures and images for: Fenoldopam to prevent acute kidney injury after major surgery—a systematic review and meta-analysis
Source: Crit Care. 2015 Dec 25;19:449. doi: 10.1186/s13054-015-1166-4 (PMC4699343; doi:10.1186/s13054-015-1166-4)

**Additional file 1: Funnel Plot for Primary Outcome (Author Defined Acute Kidney Injury)**


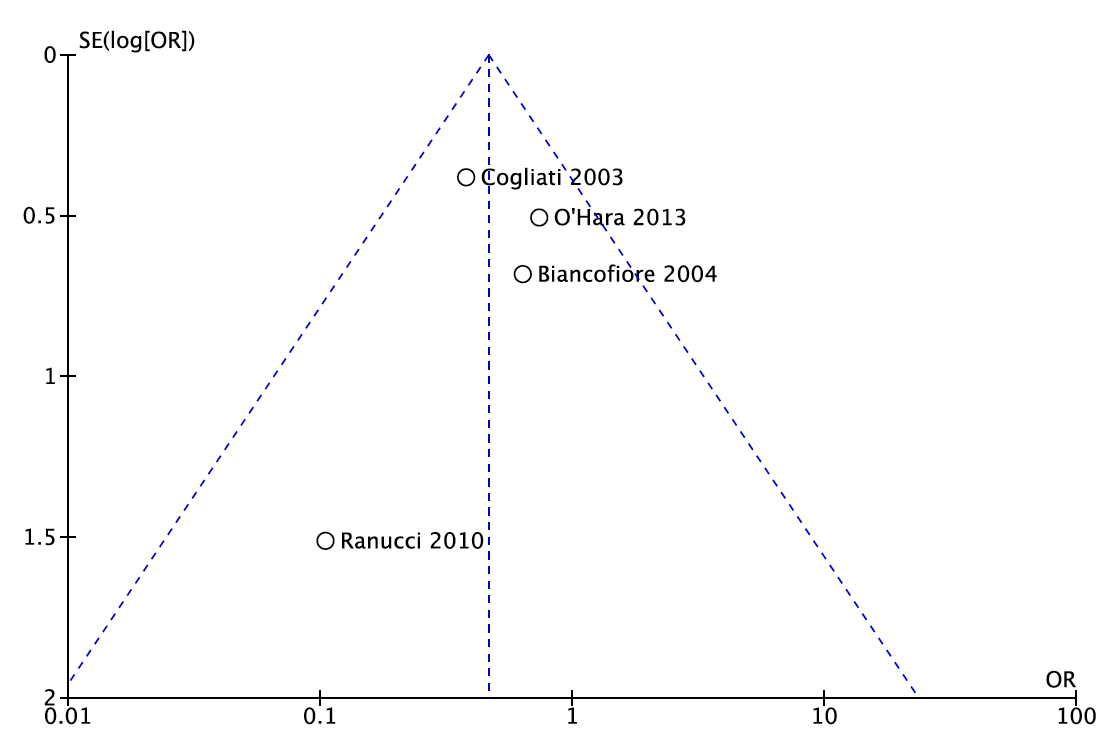

Supplement: Additional file 1: — Funnel plot for primary outcome (author defined acute kidney injury). (DOC 55 kb) [file 13054_2015_1166_MOESM1_ESM.doc]
