# Supplementary material for: Fenoldopam to prevent acute kidney injury after major surgery—a systematic review and meta-analysis
Source: Crit Care. 2015 Dec 25;19:449. doi: 10.1186/s13054-015-1166-4 (PMC4699343; doi:10.1186/s13054-015-1166-4)
Supplement: Additional file 2: — Forrest plot of acute kidney injury for cardiac surgery subgroup. (DOC 58 kb) [file 13054_2015_1166_MOESM2_ESM.doc]

**Additional file 2: Forrest Plot of acute kidney injury for cardiac surgery subgroup**


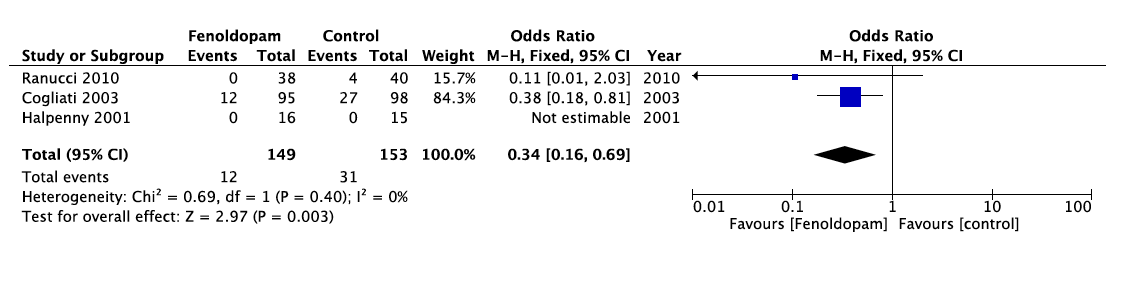


Abbreviations: CI = confidence interval
