# Supplementary material for: Fenoldopam to prevent acute kidney injury after major surgery—a systematic review and meta-analysis
Source: Crit Care. 2015 Dec 25;19:449. doi: 10.1186/s13054-015-1166-4 (PMC4699343; doi:10.1186/s13054-015-1166-4)
Supplement: Additional file 3: — Forest plot of new requirement for renal replacement therapy. (DOC 68 kb) [file 13054_2015_1166_MOESM3_ESM.doc]

**Additional file 3. Forest Plot of new requirement for Renal Replacement Therapy**


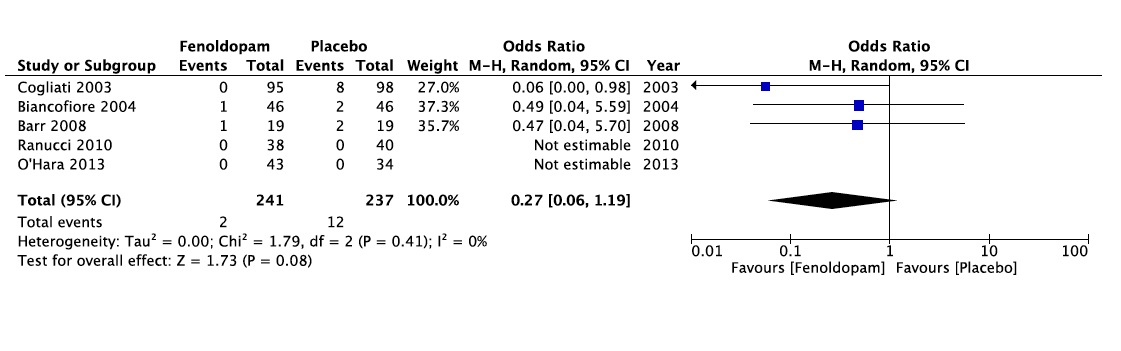


Abbreviations: CI = confidence interval
